# Serial Changes in Magnetic Resonance Imaging During the Acute Phase of Neurosyphilis With Trigeminal Nerve Palsy

**DOI:** 10.7759/cureus.78984

**Published:** 2025-02-14

**Authors:** Taiki Matsubayashi, Ryoko Muramatsu, Shuko Fujiki, Misako Furuki, Masato Obayashi

**Affiliations:** 1 Department of Neurology, National Hospital Organization Disaster Medical Center, Tokyo, JPN

**Keywords:** cerebrospinal fluid, magnetic resonance imaging, neurosyphilis, rapid plasma reagin, trigeminal nerve

## Abstract

Syphilis cases are increasing worldwide, raising concerns about a potential rise in neurosyphilis. However, neurosyphilis presenting with cranial nerve palsy as the initial symptom remains rare.

A 45-year-old man presented with fever, right-sided headache, and numbness localized to the first branch of the right trigeminal nerve for eight days. Initial brain magnetic resonance imaging (MRI), performed on day eight after symptom onset, revealed enlargement of the right trigeminal nerve. A follow-up MRI on day 15 later demonstrated a hyperintense lesion on the T2-weighted image in the pontine region adjacent to the swollen nerve. The lesion in the pons had an iso-intensity on diffusion-weighted imaging and elevated apparent diffusion coefficient (ADC) values. Cerebrospinal fluid analysis showed an elevated cell count, a positive fluorescent treponemal antibody absorption test, and increased rapid plasma reagin levels, leading to a diagnosis of neurosyphilis. Intravenous penicillin G treatment was initiated on day 15, resulting in an improvement of the patient's fever, headache, and numbness. Additionally, a follow-up brain MRI on day 44 showed reduced trigeminal nerve swelling and resolution of signal changes in the brain parenchyma.

The chronological progression observed on MRI, with signal changes in the adjacent brain parenchyma appearing after trigeminal nerve swelling, and the signal changes in the brain parenchyma accompanied by elevated ADC values, suggest that the abnormal signal changes in neurosyphilis may be linked to angiogenic edema resulting from secondary inflammation. In this case, early diagnosis likely contributed to a favorable therapeutic response. This case further highlights the importance of considering neurosyphilis in the differential diagnosis of patients presenting with trigeminal nerve impairment.

## Introduction

Neurosyphilis is a complication resulting from the invasion of *Treponema pallidum* into the central nervous system, characterized by a wide spectrum of symptoms [1]. In recent years, the incidence of syphilis has been rising in various countries [2,3], raising concerns about an associated increase in neurosyphilis cases. Neurosyphilis is classified into early and late stages, with early cases often presenting asymptomatically [3]. In contrast, symptomatic syphilitic meningitis can manifest with various symptoms, including headache, confusion, and seizures [4], though cranial nerve palsy is rarely the initial presentation [5]. Among cranial nerve palsies presenting as initial symptoms of neurosyphilis, involvement of the oculomotor, facial, and vestibulocochlear nerves is the most common, whereas trigeminal nerve palsy is a rare initial manifestation [5].

Although signal changes in the brain parenchyma on magnetic resonance imaging (MRI) are known to occur in neurosyphilis [6], their underlying mechanisms remain unclear. Here, we present a case of syphilitic meningitis complicated by trigeminal neuropathy, characterized by a hyperintense lesion on MRI in the right pons that developed following enlargement of the right trigeminal nerve. This case features disease progression documented through serial MRI follow-up, offering valuable insights into the course of early neurosyphilis.

## Case presentation

A 45-year-old man had a history of syphilis two years earlier. He initially had presented with a genital rash and was diagnosed with early syphilis, for which he received a single intramuscular injection of penicillin G. Following treatment, the rash resolved, and a decrease in serum rapid plasma reagin (RPR) levels was confirmed, indicating the completion of therapy.

Eight days before the visit to our hospital, he presented with persistent pain around his right temple. He experienced severe pain with minimal touch to the temporal region, exhibiting sensory hypersensitivity. The day after symptom onset, he visited a local physician, and a head computed tomography (CT) scan showed no abnormalities. However, numbness around his right eye and fever developed on the same day. His symptoms, including right headache, numbness around his right eye, and fever, persisted, and thus, he visited our hospital. On examination, his vital signs were as follows: body temperature, 37.3°C; blood pressure, 146/64 mmHg; heart rate, 64 beats/min; and oxygen saturation, 98% in room air. He was conscious and alert, with a Glasgow Coma Scale score of E4V5M6. No skin rash was present. A cranial nerve examination revealed dysesthesia and mild hypoesthesia to touch and pain (7/10), localized to the first branch of the right trigeminal nerve. The pupils were bilaterally round and equal in size, with brisk light reflexes observed. No findings suggestive of the Argyll Robertson pupils were noted. Motor weakness, sensory impairment in the extremities, or bladder and rectal dysfunctions were not noted. He also exhibited no signs of meningeal irritation, including nuchal rigidity, Kernig’s sign, and Brudzinski’s sign. Initially, mild cognitive impairment was suspected based on a Hasegawa Dementia Scale-Revised score of 27 and the Montreal Cognitive Assessment-Japanese version score of 25. However, further evaluation with the Wechsler Adult Intelligence Scale-Fourth Edition revealed average intelligence (Full-Scale Intelligence Quotient: 110), indicating no cognitive impairment.

**Figure 1:**
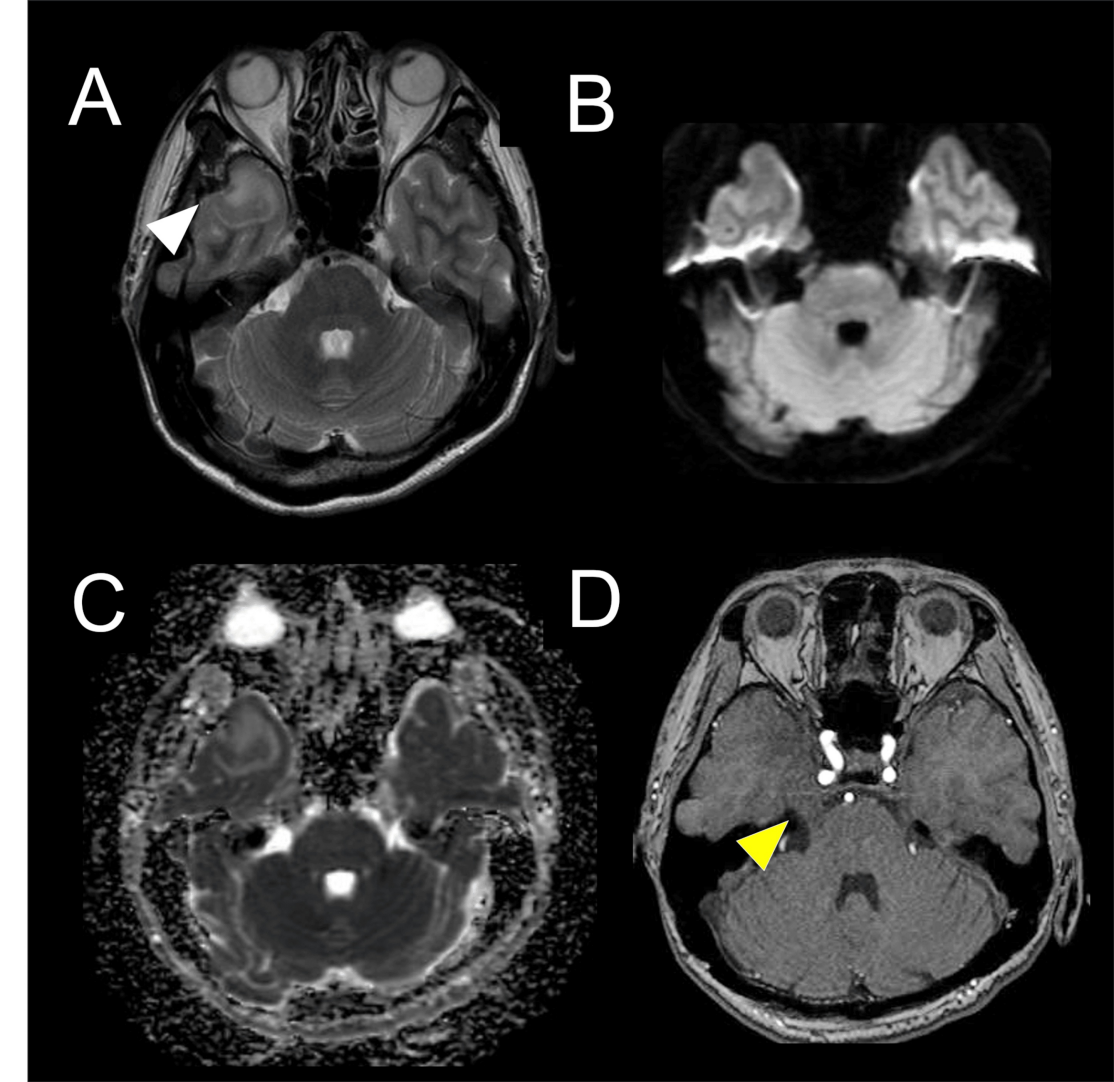
Brain magnetic resonance imaging was performed eight days after onset. T2-weighted imaging revealed a hyperintensity in the white matter of the right temporal lobe (A: white arrowhead), with an iso-intensity on diffusion-weighted imaging (B) and elevated apparent diffusion coefficient values (C). Magnetic resonance angiography revealed an enlarged right trigeminal nerve (D: yellow arrowhead).

Laboratory tests showed a white blood cell (WBC) count of 5,500/μL with 68.4% neutrophils, a C-reactive protein level of 0.09 mg/dL, and an erythrocyte sedimentation rate of 7 mm at one hour. Blood tests for infectious diseases revealed positive results for anti-Treponema pallidum antibodies with elevated RPR levels (59.2 RU). Tests for hepatitis B virus surface antigen, anti-hepatitis C antibodies, anti-human immunodeficiency virus 1/2 antibodies, anti-human T cell leukemia virus type 1 antibodies, and interferon-gamma release assays were negative. Antibodies for herpes simplex virus, varicella-zoster virus, cytomegalovirus, and Epstein-Barr virus indicated a past infection pattern. Immunological tests were negative for anti-SS-A/SS-B antibodies, anti-neutrophil cytoplasmic antibodies, anti-thyroid peroxidase antibodies, anti-thyroglobulin antibodies, and anti-aquaporin-4 antibodies. On day eight after onset, a brain MRI on T2-weighted image (T2WI) revealed a hyperintensity in the white matter of the right temporal lobe (Figure 1A), with an iso intensity on diffusion-weighted imaging (DWI) (Figure 1B) and elevated apparent diffusion coefficient (ADC) values (Figure 1C). Magnetic resonance angiography showed an enlarged right trigeminal nerve (Figure 1D).

He was admitted to our hospital on day 14. Cerebrospinal fluid (CSF) analysis showed increased WBC count (1768/3μL) with 88% lymphocytes, elevated protein level (129.3 mg/dL), decreased glucose level (46 mg/dL; CSF/serum ration 0.41), a positive result of oligoclonal bands (CSF: 20 bands, serum: none), and elevated immunoglobulin G (IgG) index (1.50). CSF examination also revealed positive results for the fluorescent treponemal antibody absorption (FTA-ABS) test (×20) and an elevated RPR level (10.7 RU). CSF bacterial cultures were negative, and cytology results were classified as class II. Laboratory results in this case were summarized in Table 1.

**Table 1:** Laboratory parameters analyzed in the serum and CSF. CMV: cytomegalovirus; CSF: cerebral spinal fluid; EBV: Epstein-Barr virus; FTA-ABS: fluorescent treponemal antibody absorption; HbA1: glycated hemoglobin; HBs: hepatitis B virus surface; HCV: hepatitis C antibodies; HIV: human immunodeficiency virus; HSV: herpes simplex virus; HTLV-1: human T cell leukemia virus type 1; RPR: rapid plasma reagain; VCA: virus capsid antigen; VZV: varicella-zoster virus

	Laboratory parameters	Value (units)	Reference value
Serum	White blood cell	5,500/μL	4,000-10,000
	Neutrophils	68.4%	40-70
	C-reactive protein	0.09 mg/dL	<0.5
	Erythrocyte sedimentation rate	7 mm at one hour	2-10
	Blood urea nitrogen	14.0 mg/dL	8-20
	Creatinine	0.95 mg/dL	0.65-1.07
	Glucose	110 mg/dL	73-110
	Anti-treponema pallidum antibodies	Positive	Negative
	RPR	59.2 RU	<1.0
	HBs antigen	Negative	Negative
	Anti-HCV antibodies	Negative	Negative
	Anti-HIV-1 and HIV-2 antibodies	Negative	Negative
	Anti-HTVL-1 antibodies	Negative	Negative
	Immunoglobulin M anti-HSV antibodies	0.15	<0.8
	Immunoglobulin G anti-HSV antibodies	55.0	<2.0
	Immunoglobulin M anti-VZV antibodies	0.13	<0.8
	Immunoglobulin G anti-VZV antibodies	32.6	<2.0
	Immunoglobulin M anti-CMV antibodies	<0.85	<0.85
	Immunoglobulin G anti-CMV antibodies	120.9	<6.0
	VCA-immunoglobulin M	< x10	< x10
	VCA-immunoglobulin G	×40	< x10
	EBV nuclear antigen	×40	< x10​​​​​​​
	Interferon-gamma release assay	Negative	Negative
	Anti-SS-A/SS-B antibodies	Negative	Negative
	Anti-neutrophil cytoplasmic antibodies	Negative	Negative
	Anti-thyroid peroxidase antibodies	Negative	Negative
	Anti-thyroglobulin antibodies	Negative	Negative
	Anti-aquaporin-4 antibodies	Negative	Negative
	HbA1c	5.4%	4.9-6.0
CSF	Color	Clear	-
	White blood cell	1,768/3 μL	0-15
	Lymphocytes	88%	40-80
	Protein	129.3 mg/dL	15-45
	Glucose	46 mg/dL	50-70
	Immunoglobulin G index	1.5	<0.7
	FTA-ABS	×20	Negative
	RPR	10.7 RU	<1.0
	Oligoclonal bands: 20 bands in CSF and none in serum	Positive	Negative

**Figure 2:**
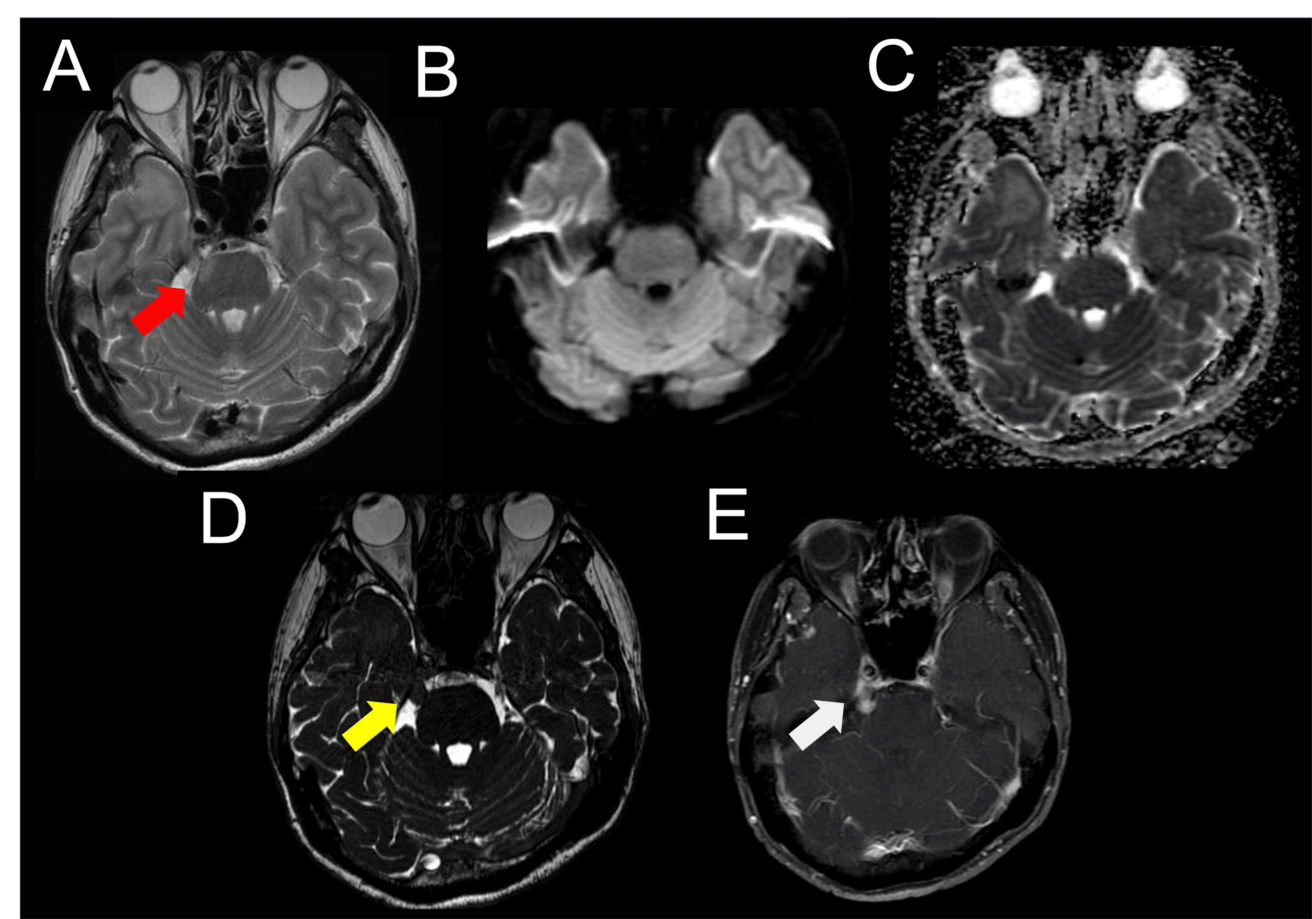
Brain magnetic resonance imaging was performed 15 days after onset. T2-weighted imaging revealed hyperintensity in the white matter of the right temporal lobe and the right pons (A: red arrow). The lesion in the right pons had an iso intensity on diffusion-weighted imaging (B) and elevated apparent diffusion coefficient values (C). An enlarged and contrast-enhanced right trigeminal nerve was observed on fast imaging employing steady-state acquisition sequences (D: yellow arrow) and contrast-enhanced T1-weighted sequences (E: white arrow), respectively. No contrast enhancement was observed in the pontine lesion.

On day 15, the patient continued to experience fever, headache, and trigeminal nerve impairment; however, no new cranial nerve palsies emerged. A brain MRI on day 15 revealed a new hyperintense lesion in the right pontine region on T2WI (Figure 2A) with iso-intensity on DWI (Figure 2B) and elevated ADC values (Figure 2C). MRI further revealed persistent enlargement of the right trigeminal nerve on fast imaging employing steady-state acquisition sequences (Figure 2D), accompanied by contrast enhancement (Figure 2E). No contrast enhancement was observed in the pontine lesion. A contrast-enhanced CT scan of the trunk revealed no evidence of malignancy.

**Figure 3:**
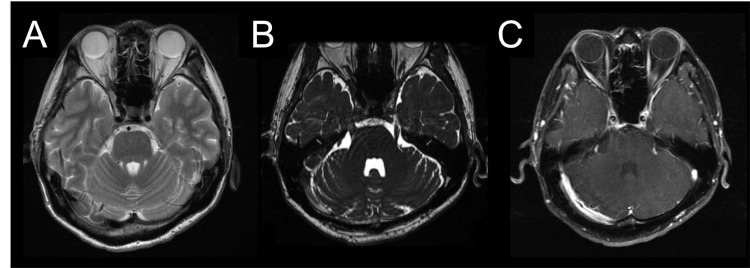
Brain magnetic resonance imaging was performed 44 days after onset. T2-weighted imaging demonstrated the resolution of the hyperintensity in the white matter of the right temporal lobe and pons (A). Fast imaging employing steady-state acquisition sequences revealed a reduction in the size of the right trigeminal nerve (B), and contrast-enhanced T1-weighted imaging showed no enhancement of the right trigeminal nerve (C).

He was suspected of having syphilitic meningitis as an early stage of neurosyphilis and was started on intravenous penicillin G (24 million units/day) on day 15. After the initiation of antibiotic treatment, no signs suggestive of the Jarisch-Herxheimer reaction, such as fever, headache, or other neurological impairments, were observed. During the antibiotic treatment period, oral probenecid was co-administered. The right-sided headache, numbness around his right eye, and fever resolved within two days of starting treatment. He was discharged after 14 days of antibacterial therapy. No additional therapy was administered after the completion of the 14-day antibiotic treatment. Follow-up CSF analysis on day 44 revealed a decreased WBC count (153/μL), a reduced protein level (40.4 mg/dL), and a slight decline in the IgG index (1.26). A follow-up MRI on day 44 after onset showed the disappearance of hyperintense lesions in the right pontine region and temporal lobe white matter (Figures 3A, 3B), as well as the resolution of contrast enhancement and enlargement of the trigeminal nerve (Figure 3C). CSF analysis on day 72 showed a significantly decreased WBC count (63/3μL) and normalization of protein level (23.9 mg/dL) and RPR (<0.5 RU). Additionally, a blood test on day 72 showed a reduction in RPR levels to 28 RU. The patient was ultimately diagnosed with neurosyphilis based on CSF findings, exclusion of other diseases, and responsiveness to antibiotic treatment.

## Discussion

In this case, brain MRI images were monitored during the acute phase of syphilitic meningitis with trigeminal nerve palsy. Among patients with neurosyphilis presenting with an isolated trigeminal nerve lesion as the initial manifestation, MRI findings have been documented in only three cases prior to this one (Table 2) [7-9]. In all cases, including the present one, trigeminal nerve involvement was unilateral, consistent with the commonly unilateral presentation observed in other cranial nerve manifestations of neurosyphilis [5]. MRI exhibited enlarged trigeminal nerves with contrast enhancement in all four patients, including the present case. However, only one other case [7], besides the present one, demonstrated signal changes in the pontine region adjacent to the thickened trigeminal nerve.

**Table 2:** Clinical and imaging features of neurosyphilis with trigeminal neuropathy. *The time from disease onset to the visit to the hospital. FLAIR: fluid-attenuated inversion recovery; MRI: magnetic resonance imaging; n.a: not available; T2WI: T2-weighted imaging

Study	Case	Age	Sex	Duration*	Clinical course	MRI findings			
						Trigeminal nerve			Parenchyma
						Side	Enlargement	Contrast effects	
Ji Hye Jang EJK et al. [7]	1	63	M	1-week	Dizziness, gait disturbance, headache, and dysarthria.	Right	+	+	Hyperintensity at right pons and right middle cerebellar peduncle on FLAIR and T2WI
Honorio GL et al. [8]	2	20	F	2-month	Right temporal headache and right trigeminal nerve palsy.	Right	Initial: + Second (after treatment): -	Initial: + Second: -	
Medeiros L et al. [9]	3	20	F	4-month	Facial paresthesia and mandible abduction difficulty.	Right	Initial: + Second (after treatment): -	Initial: + Second: -	
Our case	4	44	M	1-week	Right headache, fever, and facial numbness in the V1 region.	Right	Initial: + Second: + Third (after treatment): improved	Initial: n.a Second: + Third: -	Initial: Hyperintensity at the right temporal lobe white matter on T2WI. Second: Hyperintensity at the right temporal lobe white matter and the right pons on T2WI. Third: Disappearance of hyperintensity.

To our knowledge, this is the first reported case of neurosyphilis with a trigeminal nerve lesion in which disease progression was confirmed through serial MRI follow-up. The initial MRI revealed an enlarged right trigeminal nerve without abnormalities in the pontine region, whereas a follow-up MRI demonstrated a hyperintense lesion in the right pontine area adjacent to the trigeminal nerve. Several mechanisms have been proposed to explain the abnormal signal changes in the brain parenchyma on MRI in neurosyphilis. It is hypothesized that mixed edema arises through the following processes: angioedema caused by inflammation of meningeal vessels; cellular edema resulting from hypoxia in the brain parenchyma due to small-vessel obstruction; and interstitial edema caused by obstruction of arachnoid villi associated with fibrin and leukocyte deposition [10]. In this case, parenchymal MRI changes in the pons appeared following trigeminal nerve swelling, suggesting that the abnormal MRI signals were due to secondary inflammation. Elevated ADC values on MRI further pointed to angioedema as the most likely pathogenesis. Consistent with previous reports of reversible MRI signal changes, the rapid resolution of MRI signal changes in the brain parenchyma, including the temporal lobe white matter and pontine region, following antibacterial therapy supports the hypothesis that the underlying pathophysiology is an edematous condition [11,12]. Consequently, MRI follow-up in this case indicates that angiogenic edema caused by secondary inflammation is a key mechanism underlying MRI signal changes in neurosyphilis.

The Venereal Disease Research Laboratory (VDRL) test of CSF is recommended for the diagnosis of neurosyphilis due to its high specificity [13]. However, the CSF VDRL test could not be performed in Japan, including this case. Moreover, the CSF VDRL test is known to have low sensitivity [3], meaning that a negative result does not rule out neurosyphilis. When CSF VDRL is negative, the CDC guidelines recommend considering additional CSF findings, such as WBC, protein levels, and FTA-ABS results [14]. Additionally, the CSF RPR test has been reported to have relatively high specificity [4]. In the UK guidelines, a positive CSF RPR result is considered equivalent to a positive CSF VDRL result [15], making it a valuable diagnostic tool when VDRL testing is unavailable. In this case, CSF analysis revealed elevated RPR levels, increased WBC and protein levels, and a positive FTA-ABS test, strongly supporting the diagnosis of neurosyphilis. This case demonstrated positive oligoclonal bands and an elevated IgG index, which are common yet nonspecific findings associated with neurosyphilis [16]. The diagnosis was further confirmed by excluding other potential diseases and observing significant clinical, CSF, and imaging improvements following treatment with antibacterial therapy. This case suggests the importance of considering neurosyphilis in the differential diagnosis of patients presenting with trigeminal nerve impairment.

In this case, penicillin G was administered as recommended by current guidelines for the treatment of neurosyphilis [15,17]. Probenecid was used to enhance the concentration of penicillin in the CSF [18]. We completed the 14-day antibiotic treatment for neurosyphilis as recommended by international guidelines [3], and no additional therapy was administered. The primary goals of treatment are to prevent the progression of neurological impairment and to improve symptoms [4]. Clinical symptoms of this patient rapidly resolved after treatment initiation and treatment efficacy was further supported by improvements in imaging findings, a significant reduction in CSF WBC count, and decreased RPR in serum and CSF. These findings suggest that the treatment response in neurosyphilis should be comprehensively evaluated, primarily based on clinical symptoms. In neurosyphilis, the Jarisch-Herxheimer reaction has been reported to manifest as new neurological abnormalities, such as hallucinations, altered consciousness, or seizures [19]. In this patient, no findings suggestive of a Jarisch-Herxheimer reaction were observed following the initiation of penicillin treatment. The frequency of the Jarisch-Herxheimer reaction in neurosyphilis has been reported to range from 8% to 75%, indicating a wide variation [19]. This suggests that the reaction does not necessarily occur in the majority of patients with neurosyphilis.

Interestingly, the treatment approach for early syphilis two years earlier was also in accordance with current guidelines [20]. However, it has been reported that 25-35% of patients with early syphilis have asymptomatic neurosyphilis before receiving antibiotic treatment [4]. This indicates that standard treatment for early syphilis may not be sufficient to prevent neurosyphilis. This case highlights the possibility that neurosyphilis can still develop despite appropriate treatment for early syphilis.

In the treatment of neurosyphilis, early diagnosis is crucial to enable timely therapeutic intervention. The presence of headache has been identified as a useful indicator of therapeutic response, as it may facilitate early diagnosis [21]. Headache is not a specific finding of neurosyphilis but rather a common symptom in meningitis. However, only 39% of 141 neurosyphilis patients reportedly present with headache, and fever is observed in only 23% of cases [21]. This suggests that clinical signs indicative of meningitis are often absent in syphilitic meningitis, making early diagnosis challenging. Our patient initially presented with a right-sided headache accompanied by sensory hypersensitivity but without meningeal signs, suggesting that the headache was more likely due to persistent trigeminal nerve inflammation rather than meningitis itself. The presence of a headache prompted neurological evaluations, leading to the early diagnosis of neurosyphilis and the initiation of appropriate antimicrobial therapy approximately two weeks after symptom onset. Consequently, the patient showed significant clinical improvement following treatment.

## Conclusions

We diagnosed a patient with syphilitic meningitis complicated by right trigeminal neuropathy. In this case, brain MRI images were monitored during the acute phase of syphilitic meningitis with trigeminal nerve palsy. MRI revealed parenchymal changes with elevated ADC values in the right pons that developed following swelling of the right trigeminal nerve. This case suggests that brain parenchymal signal changes in neurosyphilis may be caused by angiogenic edema resulting from secondary inflammation.

This case was comprehensively diagnosed as neurosyphilis based on CSF findings, exclusion of other diseases, and the patient’s response to antibiotic therapy. Furthermore, early diagnosis allowed for a favorable treatment response. With the global rise in syphilis cases, it is crucial to consider neurosyphilis in the differential diagnosis of patients presenting with trigeminal nerve impairment.
